# Miscellaneous

**Published:** 1884-08

**Authors:** 


DESCRIPTIVE MUSIC.—Some people can't seem to appreciate descriptive music. A Boston composer has set the roar of Niagara Falls to music. When he played the piece before a company of critics and asked them if they understood it, the man whose opinion the rest agreed with said that it was very clear to him that the waiter had fallen down stairs, upset a hat tree, broken three soup plates and a lamp, and hit his jaw a blow that broke four teeth and made him howl. But he didn't quite see what tripped the fellow at the head of the stairs. The composer at once leaped from the window and fled to the woods.

THE UTILITY OF PAIN.—The utility of pain is seen in the membrane which sweeps the surface of the eye, for instance, in several animals, whenever any irritant particle is brought into contact with these delicate structures. The pain caused by a foreign body sets up reflexly a muscular contraction in this membrane, and thus it is brought across the eye, sweeping the surface; and so the offending matter is removed. When the foreign body is too fixed to be so removed disorganization of the eye follows, and amid a general destruction of the organ the irritant matter is got rid of. Destruction of the eye in these animals would be a common occurrence if it were not for this muscular arrangement, and pain is the excitant; it is, as it were, the finger which pulls the trigger, and so the machinery already provided and prepared is set in action thereby. In man the suffering caused by a foreign body in the eye calls attention to the part and leads to its removal. If it were not for the pain so produced irremidiable mischief would often be permitted to go on unchecked, because unnoticed. Not only does pain so defend the eye from the injurious effects of foreigh bodies, but it often serves to protect the delicate organ from overwork; and where pain is so produced rest is given to the part, and recovery is instituted.

How to Remove a Tight Ring.—A novel method of effecting the removal of a ring which has become constricted around a swollen finger, or in any other similar situation, consists simply in enveloping the afflicted member, after the manner of a circular bandage, in a length of flat India-rubber braid, such as ladies make use of to keep their hats on the top of their heads. This should be accurately applied—beginning, not close to the ring, but at the tip of the finger, and leaving no intervals between the successive turns, so as to exert its elastic force gradually and gently upon the tissues underneath. When the binding is completed the hand should be held aloft in a vertical position, and in a few minutes the swelling will be perceptibly diminished. The braid is then taken off and immediately reapplied, when, after another five minutes, the finger, if again rapidly uncovered, will be small enough for the ring to be removed with ease.

Poison in Dyes.—A well-known physician, writing of the wearing of garments next the skin colored with aniline dyes, says that often the skin becomes covered with successive crops of small vessicles filled with a watery fluid, which, rupturing, keep the skin constantly moist. The frequent scratching demanded to relieve the almost incessant itching soon tears off the tender cuticle and causes bleeding. If the use of the aniline-dyed garments be continued, the disease increases and the skin becomes thickened and brittle, being liable to form painful fissures, having indurated edges that bleed readily. If the color of any bright-colored undergarment be readily removed by soap and water, the dye is of a very low grade, and being freely soluble, is all the more pernicious. When a bit of any suspected scarlet garment is washed in water of ammonia, the color will be dissolved if it is cochineal, but if it be aniline it will not be dissolved. In the former case the garment may be safely worn ; in the latter it should be avoided like any other poison.

---

SUCCESS.—Every one must patiently bide his time ; not in idleness, in useless pastime, or qerulous dejection, but in constantly accomplishing his task, that when occasion comes he may be equal to it. The talent of success is nothing more than doing what you can do well, without a thought of fame. If it comes at all it will come because it is deserved, not because it is sought after. It is a very indiscreet and troublesome ambition, which cares so much what the world says of us, to be always anxious about the effect of what we do or say ; to be always shouting to hear the echo of our own voices.

---

CLEANLINESS OF SINKS.—One of the most prolific causes of defilement and offensive odors in kitchen sinks and their outlets is the presence of decaying grease. This comes from the emptyings of kettles in which meat has been cooked, in the dish water, and in the soap The grease lodges in every crevice and catches at every obstruction. A remedy may be found in the use of the common alkalies instead of soap, aqua ammonia in washing clothes, and borax in washing lawns and laces, and washing soda in cleaning dishes. These alkalies prevent a solid fat from forming in the sink and its pipes, and neutralize all effects of decomposing fat.

---

The difference between cut and cube sugar is considerable ; the cut sugar, which is somewhat rough on the surface being pure loaf sugar cut into lumps. It commands a higher price in the market, and cannot be adulterated. It is called in the market "cut loaf." The other quality is what is known as "cubes." The cut-loaf sugar is made in lumps of fifty pounds out of cane sugar, then sawed into slabs, and these slabs are partly cut through and partly broken. It is easy to distinguish the marks of cutting and breaking on each lump. The cube sugar is made of soft sugar and pressed in moulds, which gives the smooth appearance. The cut-loaf sugar will keep its shape in any climate, and is suitable for shipment. The cube sugar will sometimes on a sea voyage resume the consistency of soft sugar, and the change of form is due to adulteration. The safest sugar for any one to buy is pure loaf sugar, and it is much sweeter than any other. The principal substance used in adulterating sugar is glucose. While glucose is sweet, it is easily detected by the expert because it is not so sweet as cane sugar. It is, nevertheless, very extensively used to adulterate cane sugar, and produce the cheap sugars which are sold in the market. The glucose is not harmful as food, but its sweetening properties are limited.

"Papa," asked a little boy, looking up from his Sunday-school lesson, "what are 'the wages of sin'?" "The wages of sin these days," replied the old man, earnestly, "depend upon circumstances and one's opportunities and business capacity. But they run up into thousands, my boy; they run up into thousands."

Why, in sea-bathing, is the water warm when the wind is from the sea toward the shore? An old salt says that the answer is simple enough. When the wind is from the sea the surface water people bathe in, which is warm, is blown and held in shore; when the wind is off shore the surface water is blown out, and cold water from the depths below the surface takes its place.

Every religion has its day. What Sunday is to the Christians Monday is to the Greeks, Tuesday to the Persians, Wednesday to the Assyrians, Thursday to the Egyptians, Friday to the Turks. and Saturday to the Jews.

Quassia Chips in Beer.—In the neighboring town of New Britain there is a factory for the production of quassia cups—the quassia wood being so intensely bitter that a cup of fresh water, if it is a quassia cup, will become very bitter in one minute, and these cups long have been in use in some families for this tonic quality they impart to water. The chips and shavings in the cup factory were thrown away or burned, until some of the lager beer brewers discovered that they were available in the place of hops for lager beer; then a demand arose for them, until now the proprietor of the shop is making more money out of his chips and shavings than he is making out of his cups.—[*Hartford Times.*

Unalterable Paste.—Take one tablespoonful of flour, add gradually one pint of cold water; boil slowly and stir well to prevent burning until it thickens; keep boiling till it becomes thin, add one teaspoonful of nitromuriatic acid, and boil till it again thickens, when it is ready for use. This paste is harmless, cheap and will neither turn sour nor mould.—[*Scientific American.*

